# Supplementary material for: End Sequence Analysis Toolkit (ESAT) expands the extractable information from single-cell RNA-seq data
Source: Genome Res. 2016 Oct;26(10):1397–410. doi: 10.1101/gr.207902.116 (PMC5052061; doi:10.1101/gr.207902.116)
Supplement: Supplemental Material [file supp_gr.207902.116_Supp_Materials.tar.gz › ESAT-esat_v0.1/doc/html/classjaligner_1_1util_1_1_commons.html]

ESAT: jaligner.util.Commons Class Reference


ESAT


- Main Page
- Related Pages
- Packages
- Classes
- Files

- Class List
- Class Index
- Class Hierarchy
- Class Members

All Classes Namespaces Files Functions Variables Enumerator Pages

- jaligner
- util
- Commons

Static Public Member Functions |
Static Public Attributes |
List of all members

jaligner.util.Commons Class Referenceabstract

|  |  |
| --- | --- |
| Static Public Member Functions | |
| static String | getFileSeparator () |
|  | |
| static String | getLineSeparator () |
|  | |
| static String | getUserDirectory () |
|  | |
| static void | setJnlp (boolean jnlp) |
|  | |
| static boolean | isJnlp () |
|  | |
| static String | getCurrentRelease () |
|  | |
| static String | getJAlignerInfo () |
|  | |

|  |  |
| --- | --- |
| Static Public Attributes | |
| static final String | TAB = "\t" |
|  | |

## Detailed Description

Global constants/variables/settings

Author
:   Ahmed Moustafa (ahmed.nosp@m.@use.nosp@m.rs.sf.nosp@m..net)

## Member Function Documentation

|  |  |  |  |  |
| --- | --- | --- | --- | --- |
| static String jaligner.util.Commons.getCurrentRelease | ( |  | ) |  |

static

Returns the current release version of JAligner

Returns
:   current release

Here is the caller graph for this function:

|  |  |  |  |  |
| --- | --- | --- | --- | --- |
| static String jaligner.util.Commons.getFileSeparator | ( |  | ) |  |

static

Returns system file separator.

Returns
:   file separator

|  |  |  |  |  |
| --- | --- | --- | --- | --- |
| static String jaligner.util.Commons.getJAlignerInfo | ( |  | ) |  |

static

Returns information about JAligner

Returns
:   information about JAligner

Here is the call graph for this function:

|  |  |  |  |  |
| --- | --- | --- | --- | --- |
| static String jaligner.util.Commons.getLineSeparator | ( |  | ) |  |

static

Returns system line separator.

Returns
:   line separator

|  |  |  |  |  |
| --- | --- | --- | --- | --- |
| static String jaligner.util.Commons.getUserDirectory | ( |  | ) |  |

static

Returns user's current directory.

Returns
:   user's current directory

|  |  |  |  |  |
| --- | --- | --- | --- | --- |
| static boolean jaligner.util.Commons.isJnlp | ( |  | ) |  |

static

Returns true if jnlp is enabled

Returns
:   true if jnlp is enabled, otherwise returns false

|  |  |  |  |  |  |
| --- | --- | --- | --- | --- | --- |
| static void jaligner.util.Commons.setJnlp | ( | boolean | *jnlp* | ) |  |

static

Sets the JNLP flag to true of false

Parameters
:   |  |  |
    | --- | --- |
    | jnlp | true or false |

## Member Data Documentation

final String jaligner.util.Commons.TAB = "\t"

static

Tab

---

The documentation for this class was generated from the following file:

- java/jaligner/util/Commons.java


---

Generated on Fri Aug 15 2014 15:11:06 for ESAT by  

 1.8.7
